# Advancements in automated nuclei segmentation for histopathology using you only look once-driven approaches: A systematic review

**DOI:** 10.1016/j.compbiomed.2025.110072

**Published:** 2025-03-25

**Authors:** Shyam Sundar Debsarkar, Bruce Aronow, V.B. Surya Prasath

**Affiliations:** a Division of Biomedical Informatics, Cincinnati Children’s Hospital Medical Center, OH, 45229, USA; b Department of Pediatrics, College of Medicine, University of Cincinnati, OH, 45257, USA; c Department of Biomedical Informatics, College of Medicine, University of Cincinnati, OH, 45267, USA; d Department of Computer Science, University of Cincinnati, OH, 45221, USA

**Keywords:** You only look once, Convolutional neural network, Deep learning, Nuclei detection, Nuclei segmentation, Histopathology, Review

## Abstract

Histopathology image analysis plays a pivotal role in disease diagnosis and treatment planning, relying heavily on accurate nuclei segmentation for extracting vital cellular information. In recent years, artificial intelligence (AI) and in particular deep learning models have been applied successfully in solving computational pathology image analysis tasks. The You Only Look Once (YOLO) object detection framework, which is based on a convolutional neural network (CNN) architecture has gained traction across various domains for its real-time processing capabilities. This systematic review aims to comprehensively explore and evaluate the advancements, challenges, and applications of YOLO-based methodologies in nuclei segmentation within the domain of histopathological images. The review encompasses a structured analysis of recent literature, focusing on the utilization of YOLO variants for nuclei segmentation. Key methodologies, training strategies, dataset specifics, and performance metrics are evaluated to elucidate the strengths and limitations of YOLO in this context. Additionally, the review highlights the unique characteristics of YOLO that enable efficient object detection and delineation of nuclei structures, offering a comparative analysis against traditional segmentation approaches. This systematic review underscores the promising outcomes achieved through YOLO-based architectures, emphasizing their potential for accurate and rapid nuclei segmentation. Furthermore, it identifies persistent challenges such as handling variances in nuclei appearances, optimizing model architectures for histopathological images, and improving generalization across diverse datasets. Insights derived from this review can provide a foundation for future research directions and enhancements in nuclei segmentation methodologies using YOLO within histopathology, fostering advancements in disease diagnosis and biomedical research.

## 1. Introduction

In recent years, the field of histopathology has witnessed remarkable advancements in automated image analysis techniques [1], particularly in the area of nuclei segmentation [2]. Nuclei segmentation plays a crucial role in various histopathological analyses, including cancer diagnosis, tumor grading, and treatment planning. Accurate nuclei segmentation is a fundamental task in digital pathology, enabling analysis of cell nuclei within histopathological images which helps in understanding tissue structure and different disease diagnosis. Over the years nuclei segmentation methods have been evolved significantly due to the advancements in computer vision and machine learning technologies. Traditional segmentation techniques include thresholding methods [3], contour-based approaches [4], machine learning models that typically depend on hand-tuned features [5]. Despite the significant advancements in nuclei segmentation, several challenges can persist in order to get the optimal performance. One of the main challenges is to handle inherent variability of in histopathological images, such as variations in straining intensity, tissue morphology, and cellular density. Additionally, nuclei segmentation may be hindered by the presence of overlapping or touching nuclei, as well as artifacts such as staining artifacts and tissue folds. Moreover, the generalization of segmentation models across different tissue types and staining protocols remains a formidable challenge, requiring robust feature representation and domain adaptation techniques. Further, the traditional methods do not generalize well across histopathology datasets necessitating various modifications. Traditional manual methods for nuclei segmentation are typically time-consuming, labor-intensive, and prone to inter-observer variability and automatic nuclei segmentation methods are desirable. With the advancement of deep learning techniques and the availability of large-scale annotated datasets, a lot of scopes have been created to counter these challenges and to enhance performance in nuclei segmentation.

Deep learning is now widely used in various challenging biomedical imaging problems such as segmentation, classification [6]. The introduction of neural network-based deep learning models offers unprecedented accuracy and robustness to improve the nuclei segmentation process. Especially, convolutional neural networks (CNNs) have emerged as powerful nuclei segmentation tool by leveraging hierarchical feature representations to capture intricate nuclear morphology and spatial relationships. These deep learning models have demonstrated remarkable performance in nuclei segmentation tasks, surpassing the capabilities of traditional methods and preparing the platform for automated histopathological data analysis at scale. While many deep learning models, such as U-Net [7], Mask R-CNN [8], and more recent transformer-based models [9], have shown promising results in nuclei segmentation, our review focuses on the You Only Look Once (YOLO) architecture due to its unique strengths. YOLO offers exceptionally high speed and efficiency by detecting and classifying objects in a single forward pass. It is particularly suitable for real-time applications of large-scale histopathological analysis. Although models like Hover-Net [10] and transformers may achieve higher segmentation accuracy, their slower inference times make them less ideal for settings where rapid analysis is critical. YOLO, first introduced by Redmon et al. [11], revolutionized object detection by proposing a single-stage, end-to-end CNN architecture. Unlike traditional two-stage methods, YOLO processes the entire image in a single pass, making it exceptionally fast and suitable for real-time applications. This efficiency makes YOLO an ideal candidate for nuclei segmentation tasks in histopathology, where large-scale image datasets require rapid and accurate analysis. YOLO’s unique architecture divides the input image into a grid and predicts bounding boxes and class probabilities directly. This enables precise localization and classification of nuclei within histopathology images. Moreover, YOLO’s ability to handle multiple object sizes, aspect ratios, and classes makes it well-suited for the diverse and complex nature of nuclei in histopathological specimens. In addition to these advantages, since nuclei segmentation is a precursor for downstream tasks such as feature extraction, classification etc., YOLO-based approaches obtain good or on-par with specialist deep learning nuclei segmentation approaches when applied to gigapixel sized histopathology whole slide images (WSIs). As a result, YOLO has gained traction in the histopathology community as a powerful tool for automating nuclei segmentation tasks. Additionally, hybrid approaches combining YOLO with CNN backbones or transformers have the potential to enhance segmentation performance utilizing YOLO’s fast inference. YOLO enables the precise localization of nuclei within histopathology images as well as captures the multiscale nature of nuclei across histopathology datasets. This enables the hybrid approaches to perform downstream tasks such as classification. Further, YOLO provides fast, reliable, and accurate multiclass segmentation results which are easily integrated into more complex pipelines. Hence, in this review apart from assessing YOLO in its pure form, focusing on its real-time processing strengths, current limitations, as well as hybrid approaches that could further improve nuclei segmentation in histopathology using YOLO-based localization along with other deep learning models.

In the field of computational histopathology, automatic image analysis is an important aspect, and nuclei segmentation is one of the important tasks in automated pathology pipelines. The grid-centric approach of YOLO allows it to recognize detailed spatial relationships and detect nuclei at multiple scales in a robust manner. The key novelty of the YOLO model is the introduction of single forward pass to predict bounding boxes, class probabilities and segmentation masks. In this systematic review, we aim to provide a comprehensive overview of advancements in automated nuclei segmentation in histopathology using YOLO. By synthesizing findings from a wide range of studies, we seek to elucidate the strengths, limitations, and potential applications of YOLO in nuclei segmentation in histopathology image data. We succinctly describe YOLO and the evolution of its variants along with their advantages, weakness, and characteristics. Furthermore, our review explores the various histopathological nuclei specific datasets, methodologies, evaluation metrics, and challenges associated with YOLO-based nuclei segmentation approaches. Through this systematic review, we aim to provide valuable insights into the current state-of-the-art in automated nuclei segmentation and offer recommendations for future research directions in this rapidly evolving field.

The rest of our review is organized as follows. Section 2 sets the background and the evolution of YOLO models. Section 3 provides the details of the systematic review, datasets, and metrics along with detailed results. Section 4 concludes the paper with a discussion of challenges and directions.

## 2. Background

### 2.1. Object detection, segmentation and classification

Medical imaging is an important part in advanced diagnosis of different chronic diseases nowadays. It contributes to monitoring treatment responses and gaining insights into tissue structures as well. Understanding cellular structures and finding anomalies perform key part of the image analysis. Nuclei analysis stands out as a crucial domain in the medical imaging field, unlocking valuable information about tissue and cellular structures and abnormalities. Integration of detection, segmentation and classification processes covers the detailed analysis of complex nuclei and cell structures unravelling the intricate details present within images.

- Detection: Nuclei are usually very tiny objects within medical images (WSIs, microscopic images etc.) and provide very time-consuming job for the pathologists to identify and study the features. Automatic object detection is a process that helps pathologists in distinguishing instances of nuclei objects within the image. It identifies the nuclei among the other tissue objects and provides bounding boxes surrounding them. That helps the experts to focus only on the bounding boxes regions and ignore the other parts of the image. Usually, accuracy and time consumption are considered as effective metrics for nuclei object detection. The nuclei detection algorithms can be categorized in two major types – one stage and two stage object detectors. Whereas one stage detectors provide more time efficient solution, the two stage detectors perform more accurately [12] For two stage detectors, a separate region proposal network (RPN) is needed [13] to predict region proposals (bounding boxes) for the nuclei and the second stage classifies the region proposals. Region-based convolutional neural network (R-CNN) [12] and its different variants are popular two stage detector methods. One stage detectors do not need an extra step of RPN or the bounding boxes but apply classification directly. YOLO [11] and single shot multibox detector (SSD) [14] are two important one stage detectors.
- Segmentation: Segmentation process stands for precisely defining the boundaries of objects within an image. Here, each individual pixel is labeled to a certain category by trying to identify certain pattern from the tissue structures. The output consists of masks which define the shape of the objects and correspond to different object boundaries and regions. Nuclei segmentation stands for extracting features with more morphological details, understanding the spatial relationship and in-depth cellular analysis.
- Classification: Classification is a process wherein meaningful features are extracted from the image and recognizable patterns are identified from the features. Based on the recognized pattern the image or object is assigned to a particular class. Specifically, different regions in image are assigned to particular classes, which helps to label abnormal tissues, cells or nuclei aiding pathologists recognizing the disease [15]. Several deep learning CNN models are used as successful classifiers, such as VGG [16], ResNet [17], DenseNet [18], AlexNet [19] etc. There are traditional machine learning classifiers [20], such as – SVM, decision tree, random forest, naïve bayes, k-nearest neighbor (KNN) [21] that were used for classification as well.

Recent breakthroughs in computer vision have developed transformer-based models that demonstrated remarkable success in various medical imaging tasks, including segmentation. Models like Vision Transformers (ViT) [9] and its variants have gained high popularity due to their efficiency in capturing long-range dependencies and complex spatial relationships in images. Although they obtained promising results in histopathology [22,23], transformers’ higher computational costs and inference times make them less effective than YOLO in nuclei segmentation. YOLO is in general faster compared to other deep learning algorithms, because it processes the entire input image in a single pass through the underlying neural network, meaning it only analyzes the image once to detect all possible objects that are present in it. This contrasts with other methods that require multiple stages, making it significantly quicker for real-time object detection applications see Ref. [24] for more details about comparison of running times. This is also advantageous in large-scale histopathological image analysis pipelines where nuclei segmentation is usually followed up by downstream tasks. Hybrid approaches combining YOLO with CNN backbones [25] or transformers [26] have also been explored in other domains. These architectures utilize the speed and efficiency of YOLO’s one-stage detection while incorporating the feature extraction capabilities of CNNs or transformers. Particularly, YOLO-transformer hybrids aim to enhance the model’s segmentation accuracy by utilizing transformer layers for better contextual understanding. However, these hybrid approaches have not been applied to nuclei segmentation in histopathology. Despite the advantages of transformers in segmentation tasks, our review focuses on YOLO’s suitability for real-time applications in histopathology. Future work could explore integrating YOLO with transformers to balance speed and accuracy in nuclei segmentation, providing a promising avenue for further research.

### 2.2. Basic YOLO model and different versions

YOLO [11] revolutionized the traditional two-step process of object detection by executing both localization and classification in one task. This unique approach divides the input image into a grid and predicts bounding boxes and class probabilities directly, enabling real-time detection with exceptional accuracy. YOLO consists of a distinctive feature that predicts multiple bounding boxes per grid cell. During training only one bounding box is assigned as predictor for each object. It is achieved by designating the predictor with the highest intersection over union (IOU) with the ground truth (GT) as the “responsible” predictor. This approach encourages specialization among predictors, enabling each to excel in predicting distinct object sizes, aspect ratios, or classes. Consequently, this specialization contributes to an improved overall recall score.

An important technique employed in YOLO models is non-maximum suppression (NMS), a post-processing step crucial for improving both accuracy and efficiency in object detection. In object detection tasks, it is common that multiple bounding boxes can be generated for a single object. NMS serves to identify and eliminate redundant or inaccurate bounding boxes by refining the precision of object localization.

**Fig. 1.**
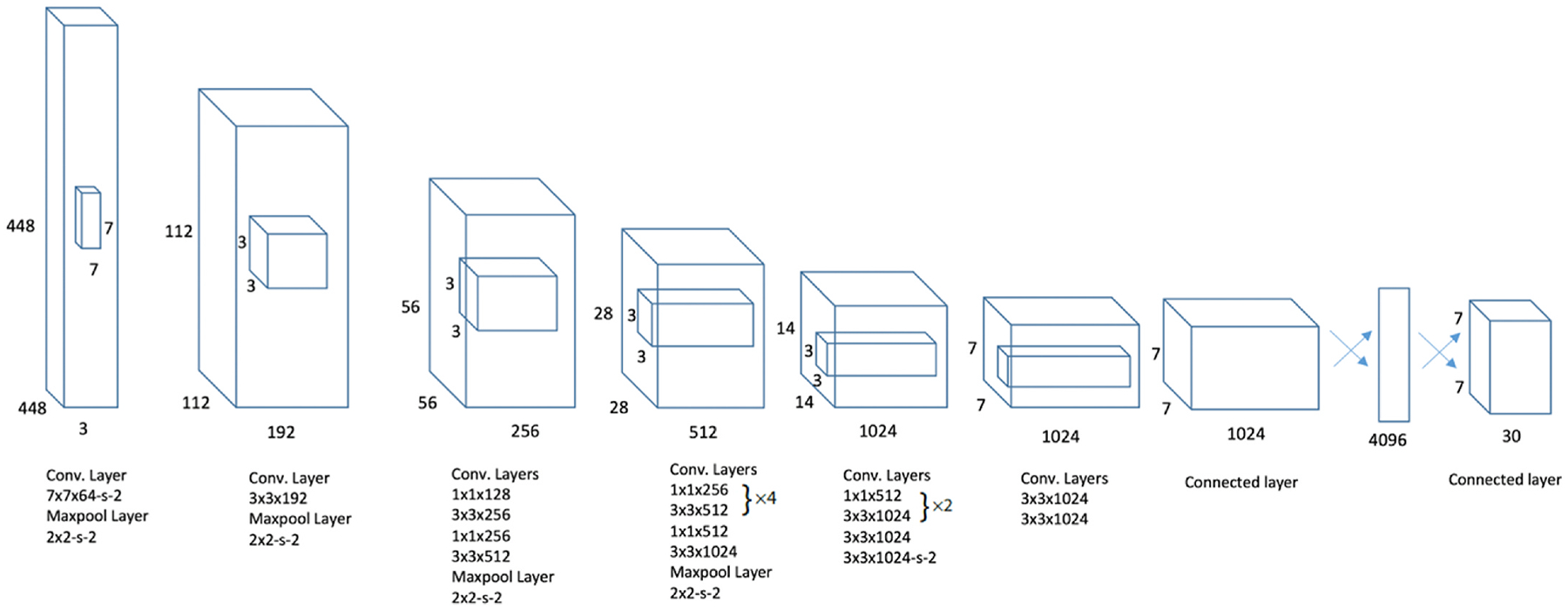
Original YOLO architecture proposed by Redmon et al. [1].

**Fig. 2.**
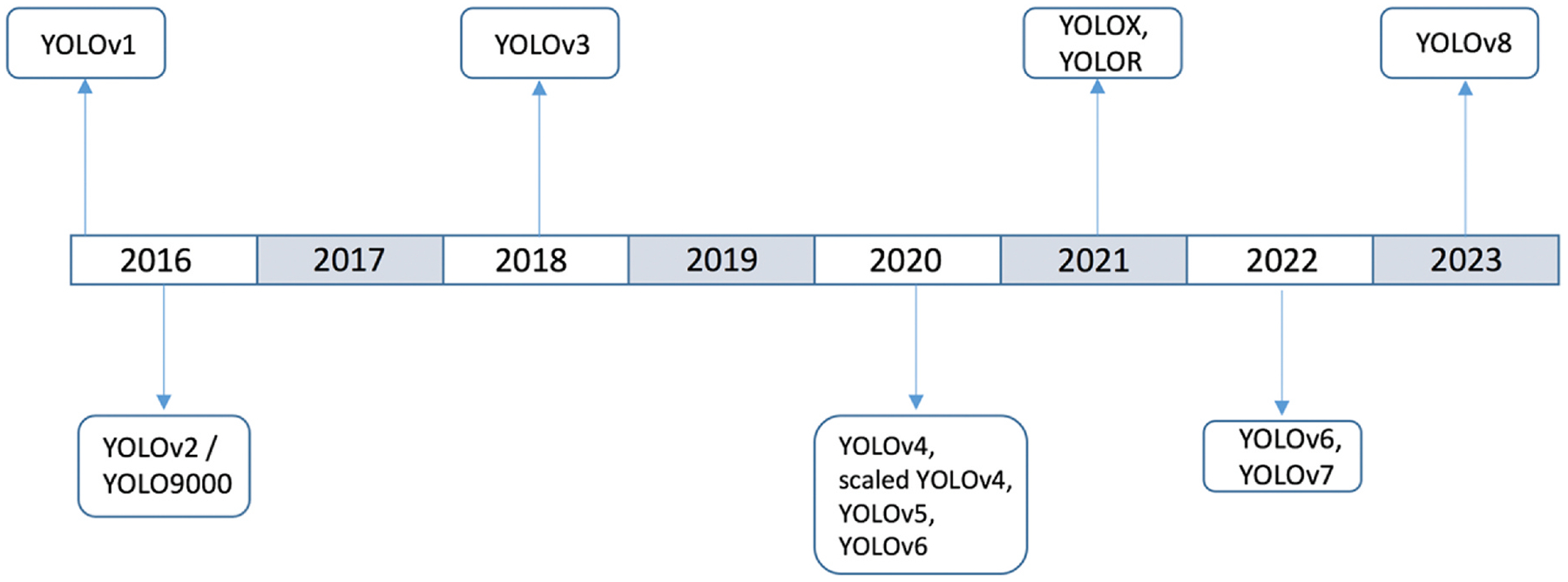
Year-wise evolution of YOLO models and their variants.

The first version of YOLO architecture consists of 24 convolutional layers followed by 2 fully connected layers as shown in Fig. 1. The first 20 convolution layers of the model were pre-trained using the ImageNet [27] dataset. This pre-training is achieved by integrating a temporary average pooling and fully connected layer. Subsequently, the pre-trained model undergoes a transformation to facilitate object detection. In the detection phase, YOLO splits the input image into an S × S grid. If the center of an object falls within a specific grid cell, that cell is specified for detecting the object. Within each grid cell, the model predicts B bounding boxes and their corresponding confidence scores. These confidence scores indicate the model’s certainty regarding the presence of an object within the box and the accuracy of the predicted box. In the following we briefly describe different versions of YOLO pertaining to the nuclei detection and segmentation works and refer the reader to the original publications for more details on the modifications. Fig. 2 shows an outline of evolution of YOLO models year by year.

#### YOLOv1:

YOLO architecture has evolved significantly since its first appearance. In 2016, the first version, referred to YOLOv1 [11], was introduced to make a significant shift in object detection processes. Its single-shot approach and grid-based prediction process made it faster than the traditional two-stage detectors. But YOLOv1 had noticeable limitations in accurately detecting small objects due to the fixed grid size and lack of a mechanism to handle different object scales. Also, it has problems in localization inaccuracies, leading to unspecific bounding box predictions. YOLOv1 used a simplified loss function – sum-squared error which did not provide optimal loss function.

#### YOLOv2:

To address issues in YOLOv1, another version from Redmon et al. [28] was proposed in 2016. This version is named YOLOv2. YOLOv2 introduced anchor boxes helping the model to predict bounding box offsets based on these anchors. This improves the model handling objects of different sizes more efficiently. YOLOv2, known as YOLO9000, introduced a new modified loss function that includes both localization loss and classification loss, leading to better overall model performance. During training phase of YOLOv2, multiple datasets were incorporated, including ImageNet and COCO [29], assisting the model generalize better to a wide range of object categories. Over 9000 different object categories detection was incorporated in YOLOv2. An average precision of 78.6 % achieved by YOLOv2 on the PASCAL VOC2007 dataset [30], outperforming its predecessor YOLOv1, which attained only 63.4 %. The YOLOv2 architecture included a backbone named DarkNet-19 [31], which consisted of 19 convolution layers and 5 max pooling layers.

#### YOLOv3:

YOLOv3 was introduced by Redmon et al. [32] in 2018 with some significant changes in the architecture to be matched with the state-of-the-art models. Like YOLOv2, here four coordinates (tx, ty, tw, and th) are predicted. But here a new objectness score prediction is incorporated for each bounding box using logistic regression [32,33]. The score is set to 1 if the bounding box has the highest overlap with a ground truth object compared to other bounding boxes. If a bounding box does not have the highest overlap but exceeds a threshold of 0.5, the prediction is ignored. The architecture introduced a new backbone network called DarkNet-53, featuring 53 convolutional layers. This enhanced model depth facilitated more advanced feature learning, resulting in improved representation of intricate patterns within the data. The architecture of YOLOv3 can be clearly described in three parts – backbone, neck and head [33]. The backbone, typically a CNN network, was used to extract essential features from the image. It captures hierarchical features at varying scales. The neck part refines, and aggregates features from the backbone, emphasizing spatial and semantic information across different scales. Additional convolutional layers, feature pyramid networks (FPN) [34], or other mechanisms may be incorporated into the neck to enhance feature representation.

#### YOLOv4:

The YOLOv4 version was released by Bochkovskiy et al., in 2020 [35]. Darknet-53 backbone was replaced with CSPDarknet53 [36], a modified and improved version. For the neck part, a modified version of spatial pyramid pooling (SPP) [37] is used over CSPDarknet53 since it notably increases the receptive field and extracts important context features without compromising the network speed. Additionally, PANet was used, instead of FPN in YOLOv3, for parameter aggregation across various backbone levels in different detector levels. As the head of the architecture, YOLOv3 (anchor based) was used. YOLOv4 used DarkNet [31] architecture along with the introduction of Mish activation function [38] for promoting smoother optimization and better generalization. A new loss function - Complete Intersection over Union (CIOU) loss [39], was introduced to get better localization accuracy. The training strategy was further improved by incorporating different data augmentation techniques and mosaic dataloading, leading to the enhancement of model’s robustness and generalization capability. For the MS COCO dataset [29] test-dev 2017, YOLOv4 showed an AP of 43.5 % and AP50 of 65.7 %, while YOLOv3 showed 33 % and 57.9 % respectively for the same dataset [35].

#### YOLOv5:

This version was released by Ultralytics in 2020 [40]. It is implemented in PyTorch boasting a wider community, more accessible resources and more user friendly. PyTorch reduced the dependency on DarkNet architectures. YOLOv5 introduced AutoAnchor algorithm, developed by Ultralytics, adjusts anchor boxes based on dataset characteristics and training settings. This applies a k-means function and a Genetic Evolution algorithm which optimize anchors with CIoU loss and Best Possible Recall as fitness functions. The network includes SPP [37] and upsample layers for multi-scale feature processing. Augmentations such as Mosaic, MixUp, and others enhance training stability. When tested on the MS COCO dataset test-dev 2017, YOLOv5x demonstrated an Average Precision (AP) of 50.7 % at an image size of 640 pixels.

#### Scaled-YOLOv4:

This version was proposed by by Wang et al. [41] in 2021, transitioning from Darknet in YOLOv4 to PyTorch. It incorporates mechanisms to adapt the depth and width of its backbone and neck networks, enabling the customization of model sizes to meet specific hardware and accuracy preferences. Two new techniques introduced are – scaling up and scaling down techniques. Scaling up involved creating a model that prioritizes accuracy even at the cost of reduced speed. Conversely, scaling down produced a model focused on speed, even at the cost of reduced accuracy. The scaled-up variant, termed YOLOv4-large model achieved a state-of-the-art 55.5 % Average Precision (AP) and 73.4 % AP50 on the MS COCO dataset at a speed of approximately 16 FPS on Tesla V100. On the other hand, the scaled down variant, termed the YOLOv4-tiny model achieved a commendable 22.0 % AP (42.0 % AP50) at an impressive speed of around 443 FPS on RTX 2080Ti. Leveraging TensorRT, a batch size of 4, and FP16-precision, the YOLOv4-tiny model achieves 1774 frames per second (FPS), showcasing its efficiency and speed for real-time applications.

#### YOLOR:

This version proposed in 2021 by Wang et al. [42] as you only learn one representation (YOLOR), this state-of-the-art object detection model stands out for its unique unified network architecture. YOLOR applied a single network for both feature extraction and prediction enhancing the efficiency during inference and potentially elevating overall performance. By leveraging explicit knowledge from shallow layers and implicit knowledge from deeper layers, YOLOR generates more comprehensive representation beyond object detection. On the MS COCO dataset test-dev 2017, YOLOR achieved an average precision (AP) of 55.4 % and an AP50 of 73.3 % while maintaining a speed of 30 frames per second (FPS) on an NVIDIA V100 graphical processing unit (GPU).

#### YOLOX:

YOLOX was proposed by Ge et al., in 2021 [43]. It was implemented using PyTorch and YOLOv3 was used as founding framework. Here a shift in approach can be seen from anchor-based to anchor-free architecture implementation, inspired by state-of-the-art anchor-free detectors like CornerNet [44], CenterNet [45], and FCOS [46]. Despite the ongoing evolution of backbones and feature pyramids in YOLO series, their detection heads have traditionally remained coupled. Where many detectors, whether one-stage or two-stage, adopted a decoupled head for classification and localization as obtaining the balance between classification and regression tasks is a well-recognized challenge. YOLOX separated these two tasks into different independent heads for each feature level leading to more precise predictions and easier optimization. To address the imbalances from anchor-free approach, the entire 3 × 3 area across the center was designated as positives. This process is known as “center sampling” strategy. YOLOX showed an optimal balance between speed and accuracy with an impressive 50.1 % AP at 68.9 % FPS on Tesla V100 GPU.

#### YOLOv6:

In 2022, YOLOv6 was introduced by Li et al. [47] to present a cutting-edge object detection model. A novel backbone architecture was used, called EfficientRep, inspired from RepVGG [48] architecture which incorporates higher parallelism and re-parameterization as compared to previous YOLO backbones. In YOLOv6, a more efficient hybrid-channel strategy for the decoupled head was introduced. Specifically, the structure was streamlined by reducing the number of middle 3 × 3 convolutional layers to just one. The width of head was jointly scaled by the width multiplier for the backbone and neck. This increases efficiency by reducing the computation cost significantly. To optimize the classifier, VariFocal loss (VFL) [49] was introduced which asymmetrically treats positive and negative samples, balancing the learning signals. For box regression loss, IoU-series loss [50] was incorporated, including generalized IoU (GIoU) [51] complete IoU (CIoU) [39], and SCYLLA-IoU (SIoU) [52]. The model achieved an impressive 57.2 % AP at approximately 29 FPS on an NVIDIA Tesla T4 when evaluated on the MS COCO dataset test-dev 2017.

#### YOLOv7:

In July 2022, the new state-of-the-art model YOLOv7 was introduced by Wang et al. [53]. They introduced specific changes in architecture and a set of “bag-of-freebies” to optimize the accuracy without compromising inference speed. The backbone is built upon efficient layer aggregation network (ELAN) [54] architecture which specifies more efficient learning without affecting the gradient part. YOLOv7 introduced an extended ELAN (E-ELAN) that applies expanding, shuffling, and merging cardinality to continually enhance the network’s learning ability without disrupting the original gradient path. YOLOv7 introduced a new model scaling approach – compound scaling. It calculates output channel changes when scaling depth, adjusting transition layers accordingly. It ensures balanced scaling for concatenation-based models, maintaining optimal structures and initial design properties. On the MS COCO dataset test-dev 2017, YOLOv7-E6 achieved an AP of 55.9 % and AP50 of 73.5 %, operated at 50 FPS on an NVIDIA V100 with the input size of 1280 pixels.

#### YOLOv8:

Ultralytics released YOLOv8 [55] in January 2023, which introduced five scaled versions to do different vision tasks like - detection, segmentation, pose estimation, tracking, and classification. The backbone is similar to YOLOv5, a modified version of cross stage partial layer (CSPLayer) is used where high-level features were merged with contextual information. This employs an anchor-free model with a decoupled head for independent processing of objectness, classification, and regression. This improves overall accuracy. Use of CIoU [39] and distribution focal loss (DFL) [56] functions enhance detection performances. On MS COCO dataset test-dev 2017, YOLOv8x achieves 53.9 % AP at 640 pixels, with a speed of 280 FPS on NVIDIA A100 GPU and TensorRT.

Table 1 succinctly summarizes the strength and weaknesses of YOLO versions. As can be seen, compared to original YOLOv1 the later versions have become faster, accurate and more efficient. We note that YOLO and its variants are widely utilized in finding objects in a variety of imaging modalities including natural images and other industrial applications [57–59]. In the field of histopathological image analysis, YOLO-based object detection and segmentation techniques have accelerated a transformative shift in the cell structure analysis, particularly in nuclei segmentation. YOLO’s approach involves unified and real-time object detection by dividing images into a grid and assigning each cell the task of detecting and segmenting nuclei. This grid-centric approach ensures a thorough analysis of the entire image, allowing the model to recognize detailed spatial relationships and detect nuclei at different scales all at once. The key novelty of the YOLO model is the introduction of single forward pass to predict bounding boxes, class probabilities and segmentation masks. YOLO’s utilization of CNN models and a specific backbone network helps in efficient features extraction, contributing significantly to understanding of various tissue structures. Apart from object detection, YOLO is capable of segmentation and classification in the context of histopathology images. Many of the computational histopathology pipelines require detection, localization and segmentation as a first step for analysis which where YOLO is proven to be useful and provides accurate results. As we discuss research works in histopathology utilizing YOLO, it is unavoidable that the continuous improvement in YOLO methodologies, including fine tuning on special histopathological dataset and integration of domain specific knowledge, contributes significantly to its efficacy in real-world scenario.

**Fig. 3.**
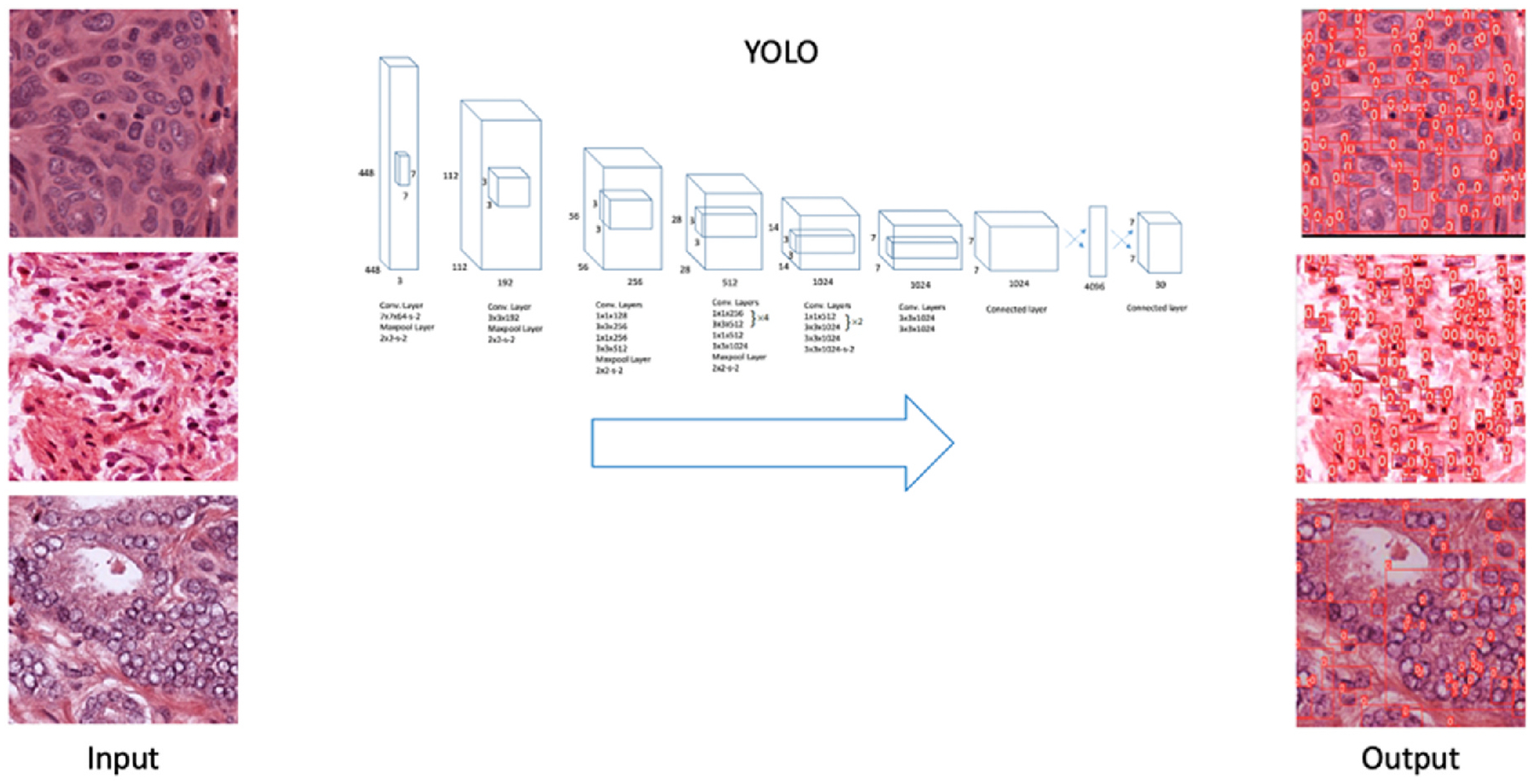
YOLO model for nuclei segmentation from histopathology images.

YOLO frames object detection from images as a regression problem to spatially separate bounding boxes and associated class probabilities in contrast to other approaches that utilize classifiers. Fig. 3 shows examples of the application of the YOLO model on histopathology images. As can be seen, the input to the YOLO model is a patch and it starts by dividing an even S × S grid, and simultaneously outputs bounding boxes and confidence in those boxes, and class probabilities. In nuclei segmentation tasks, YOLO provides precise localization through bounding boxes that act as the foundation for creating segmentation masks. Depending on the use case, the bounding boxes may be further refined into masks using post-processing techniques like pixel-based thresholding or combining YOLO with models like U-Net. Note that earlier versions like v4 outputs coarse bounding boxes unlike the newer version v8 which outputs much tighter boxes.

## 3. Systematic review of YOLO models in nuclei segmentation

### 3.1. Review search strategy and results

**Fig. 4.**
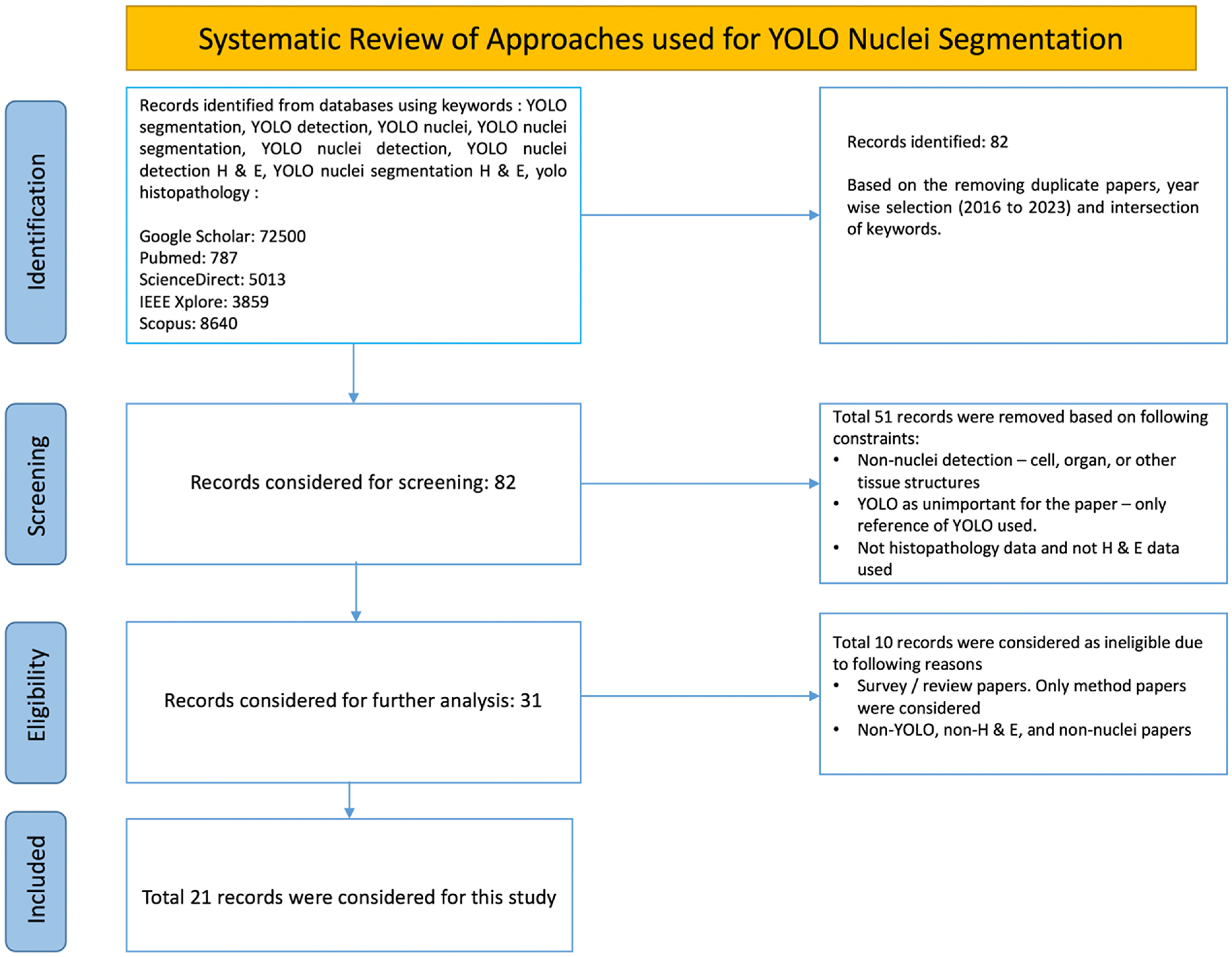
PRISMA flowchart for our systematic review.

We searched for papers in Google Scholar, PubMed, ScienceDirect, IEEE Xplore, and Scopus databases based on keywords – “YOLO based Nuclei segmentation”, “YOLO based Nuclei detection”, “YOLO based segmentation”, “YOLO based detection”, YOLO based nuclei detection on H & E data”, “YOLO in histopathology”, etc. At the primary stage we found a total of 90,799 papers. We identified a total of 82 papers after removing the duplicates, applying year-wise selection (from the inception of original YOLO model - 2016 until January 2024) and intersection of keywords. We then removed 51 papers based on three constraints – (1) they were based on cell, organ and other tissue structures and not relevant for nuclei detection, (2) YOLO was not used directly in the experiment and was considered as only reference, (3) the considered datasets were not histopathology or H & E related. Of the 31 remaining articles, 21 were further selected by removing survey/review papers and further non-YOLO, non-H & E staining and non-nuclei papers. Finally, 21 articles were considered for this review, see Fig. 4 for the preferred reporting items for systematic reviews and meta-analysis (PRISMA) flowchart.

We note that utilizing the YOLO model for nuclei segmentation has been a very vibrant research topic in recent years. The methods of applying different YOLO architectures in these works can be categorized into two main directions: (1) papers wherein only the YOLO model is used as the main segmentation or detection model and typically no changes were made within the baseline architectures, (2) papers wherein YOLO was used as a part of the whole pipeline and other ML/DL models were used as classifiers or for other steps in the overall flow. We found some histopathology nuclei segmentation works [60,61] wherein the results were compared with YOLO models. In this direction, YOLO is not directly a part of the methodology but only included as a reference.

### 3.2. Datasets and metrics

Hematoxylin and Eosin (H & E) stained whole slide images (WSIs) are very useful for understanding different morphological changes and cell structures. That is why H & E datasets are widely used for nuclei segmentation, detection and classification tasks. In this paper, we studied different datasets with H & E stained WSI slides which are widely used by different research works and relevant competitions for the advancements in deep learning methodologies to perform nuclei analysis tasks better. Here, we describe the datasets relevant for the reviewed nuclei segmentation papers. Table 2 provides a summary of information about these datasets.

- PanNuke: This dataset (https://warwick.ac.uk/fac/cross_fac/tia/dat a/pannuke) for computational pathology consisting of a total of 7904H & E-stained images with approximately 200k labeled nuclei [62]. The nuclei are categorized in 5 clinically important classes. PanNuke consists of 19 different tissue types with diverse data from various cancer types. The dataset presents the complexity and variability of real-world datasets which makes it useful for training robust AI models.
- MIDOG: This dataset (https://imig.science/midog/the-dataset) is from a MICCAI 2022 challenge called the mitosis domain generalization challenge. This dataset contains H & E images from 6 different cancer types – breast cancer, lung carcinoma, lymphoma, mast cell tumor, neuroendocrine tumor, and melanoma. In total of 9501 mitotic and 11051 non-mitotic annotations are present [63]. In a MICCAI 2021 challenge MIDOG 2021 (https://midog2021.grand-challenge.org) dataset was introduced which contains 1721 mitotic and 2714 non-mitotic annotations.
- MoNuSac: MoNuSac2020 challenge [64] (https://monusac-2020.grand-challenge.org) provided dataset of H & E-stained tissue images related to four organs and with cell types - epithelial cells, lymphocytes, macrophages, and neutrophils. The dataset was to evaluate different state-of-the-art deep learning models. The challenges included different number of annotations for different cell types. Note that this dataset was particularly aimed for nuclei segmentation and classification.
- MoNuSeg: This dataset [65] (https://monuseg.grand-challenge.org/Data) was obtained from TCGA by collecting annotated and H & E-stained tissue images from different organs with 40x magnification. The diversity of nuclei counts for different organs and its relevance in diagnosis in chronic diseases influenced the organizers to create this dataset. It was proposed during as a grand challenge in MICCAI 2018 (https://christineinspain.net/miccai2018) with the task to segment nuclei accurately based on data images diversified across a range of patients, organs, and diseases. Also, the classification of nuclei was considered as a significant challenge. The training data contained 30 images with approx. 22,000 nuclei bounding boxes while the test dataset consisted of 7000 nuclei bounding boxes.
- Atypia: This dataset (https://mitos-atypia-14.grand-challenge.org/Donwload) consists of breast cancer biopsy H&E-stained image slides. In each slide, pathologists used X20 magnification-based frames to get nuclear atypia scoring. The 20x frames were sub-divided in four 40x magnification-based frames used to annotate mitosis and to provide nuclear atypia score. Nuclei atypia scoring is a scoring system of scores 1, 2 and 3 (low, moderate or strong nuclear atypia respectively) corresponds to disparity in nuclei shapes as compared to normal nuclei [66].
- CoNSep: Colorectal nuclear segmentation and phenotypes (CoNSeP) dataset (https://warwick.ac.uk/fac/cross_fac/tia/data) was introduced by Graham et al. [10] which consisted of 41H & E-stained images with each image of 1000 × 1000×pixels and 40× magnification. The images belonged to colorectal adenocarcinoma with observed regions, such as stroma, glandular, muscular, collagen, fat and tumor regions. The dataset contained images from all major nuclei types - normal epithelial, tumor epithelial, inflammatory, necrotic, muscle, and fibroblast.
- Lizard: This dataset (https://warwick.ac.uk/fac/cross_fac/tia/data) consists of H & E-stained colon tissue images [67]. The images were collected from 6 different data sources - GlaS, CRAG, CoNSeP, DigestPath, PanNuke, and TCGA each with 20x magnification. 6 different cell classes were maintained - Epithelial, Lymphocyte, Plasma, Neutrophil, Eosinophil, Connective. The dataset was evaluated using three deep learning models U-Net [7], Micro-Net [68], and HoVer-Net [10]. Hover-Net showed the best performance.
- NuCLS: The NuCLS dataset (https://sites.google.com/view/nucls/home) [69] was introduced with the aim to work with large dataset for nuclei segmentation, and classification. It consists of around 220, 000 annotated nuclei obtained from the TCGA breast cancer dataset. The nuclei annotation was done by expert pathologists and medical personnel. More than 125,000 single-rater annotations and 97,000 multi-rater annotations were included. For single-rater data, annotations from both pathologist-reviewed and uncorrected data. Multi-rater annotations include annotations generated from both with and without suggestions from weak segmentation and classification algorithms.

We next briefly discuss some of the most important metrics relevant for nuclei detection and classification. Various metrics are considered in the literature to evaluate nuclei detection and classification algorithms by comparing their accuracy, precision, and spatial alignment with ground truth (GT) [70]. The effectiveness of an algorithm is defined by satisfactory values of metrics. The metrics’ values let researchers compare and improve models’ performances by fine tuning the approaches. We describe the standard metrics utilized by the research works covered in this review below.

- Intersection over union (IoU): IoU or Jaccard index measures overlap between the predicted mask and the GT segmentation mask. In other words, it is the intersection over union of the predicted segmentation and the GT.
$$IoU=\frac{\text{Area of Intersection}}{\text{Area of Union}}\text{or}\;IoU=\frac{TP}{TP+FP+FN}$$
- Dice coefficient or F1 score: Like IoU, it measures similarity between predicted mask and GT. However, the calculation uses 2 x intersection divided by total area of both predicted and GT.
$$\text{Dice}=\frac{2\times \text{Intersection}}{\text{Prediction}+\text{Ground truth}}\text{or}\;\text{Dice}=\frac{2TP}{2TP+FP+FN}$$
- Precision: It is the measurement of accuracy of positive predictions that indicates proportion of correctly predicted positive instances.
$$\text{Precision}=\frac{TP}{TP+FP}$$
- Recall: It indicates the capacity of measuring all the positive instances.
$$\text{Recall}=\frac{TP}{TP+FN}$$
- Accuracy: This indicates the proportion of correct predictions over all predictions.
$$\text{Accuracy}=\frac{TP+TN}{TP+FP+TN+FN}$$
  Note the notations, TP – true positive, TN – true negative, FP – false positive, and FN – false negative.
- Mean Absolute Error (MAE): This is the measurement for average absolute pairwise difference between the predicted mask and GT mask.
$$MAE=\frac{\sum |\text{Prediction}-\text{Ground truth}|}{\text{Number of Pixels}}$$
- Mean Average Precision (*mAP*): It is a very commonly used metrics for object detection and segmentation tasks. First, the average precision (*AP*) for each class based on the model prediction is calculated. Note that the *AP* is presented by the area under precision-recall curve for a particular class. Then *mAP* is calculated from the mean value of all the APs for all the classes.
$$mAP=\frac{1}{N}\sum_{i=1}^{N}AP_{i}$$
- Panoptic Quality (*PQ*): Panoptic quality can be used as nuclei segmentation and classification metrics [71].
(1) $$PQ=\frac{\sum_{\left(x_{t},y_{t}\right)\in TP}\text{IoU}\left(x_{t}y_{t}\right)}{\left|TP_{t}\right|}\times \frac{\left|TP_{t}\right|}{\left|TP_{t}\right|+\frac{1}{2}\left|FP_{t}\right|+\frac{1}{2}\left|FN_{t}\right|}$$
  Here, the first term refers to the segmentation quality and recognition quality, respectively. Mean panoptic quality,
(2) $$mPQ=\frac{1}{T}\sum_{t}PQ_{t}$$

In equation (1) in the first part, ‘*x*’ denotes a GT instance, while ‘*y*’ represents a predicted instance, with IoU (Intersection over Union) serving as the measure to determine if ‘*x*’ and ‘*y*’ uniquely match when IoU (‘*x*’,’*y*’) is greater than 0.5. This process of unique matching categorizes all instances of type ‘*t*’ within the dataset into matched pairs (TP), unmatched GT instances (FN), and unmatched predicted instances (FP). The second part represents F1 score. Equation (2) represents multiclass ‘*PQ*’ (‘*mPQ*’) averaging the ‘*PQ*’ scores across all classes.

### 3.3. YOLO as the main model for nuclei segmentation

In the field of cell biology, YOLO models can alone take the spotlight for their effectiveness in nuclei detection. Some research focuses only on nuclei detection where they use YOLO as the sole model, as shown in Table 3. Tung et al. [72] used YOLOv4 [35] model exclusively to segment nuclei from gastric cancer whole slide images (WSIs). Their study not only showcased the model’s efficiency but also demonstrated its superiority over manual pathologist work, boasting a sensitivity of 96.6 % and specificity of 89.6 %. Also, the positive predictive value (PPV) and negative predictive value (NPV) were 87.7 % and 92.5 %. The data is collected from a private database – The Taiwan Registry Database, where a total of 13,600 WSI images were collected from 50 patients of Gastric Cancer. We note that the database is private, and this makes further comparative studies harder.

Sreeraj et al. [73] followed the process nuclei atypia scoring [74] in their 2022 study, employing four YOLO architectures—YOLO-V3 [32], tiny-YOLO, YOLO-V2, and YOLO-V1 [28], in order to compare cell nuclei structures with cell structures. The MITOS-ATYPIA-14 Challenge dataset was used. YOLOv3 outperforms other models with accuracy and precision of 0.89 and 0.87, respectively. The metrics’ values for other YOLO architectures were as follows: Tiny-YOLO – accuracy 0.88, precision 0.85; YOLOv2 – accuracy 0.84, precision 0.8; and YOLOv1 – accuracy 0.81, precision 0.77.

Drioua et al. [75] applied the YOLOv5 model for nuclei detection and binary classification, utilizing the publicly available BNS dataset [76] for evaluation (https://cbio.mines-paristech.fr/~pnaylor/BNS.zip). The precision and recall values for object detection were reported as 0.86 and 0.77, respectively. The nuclei detection experiment included a comparative analysis with Mask R-CNN [8], assessing performance through IoU and F1 score metrics. The nuclei detection experiment was further analyzed by comparing the YOLOv5 results with Mask R-CNN by considering IoU and F1 score metrics. For all the test images YOLOv5 showed the best results with 0.88 F1 score and 0.93 IoU as the best values. However, the potential for exploring additional object detection models and diverse datasets could further enhance the comprehensive understanding of the methodology’s effectiveness.

Nair et al. [77] applied YOLOv4 for one step mitosis detection and classification by utilizing the dataset of MITOS-ATYPIA grand challenge-14. The training set consisted of 298H & E images with 492 annotated mitotic instances and 792 non mitotic instances. The test set consisted of 151H&E images with 219 annotated mitotic instances. The considered evaluation metrics were F1 score, recall and precision. The values for evaluation metrics for raw RGB images were 0.73, 0.85 and 0.64 respectively and for stain unmixed images [78] were 0.65, 0.60 and 0.70 respectively. The paper proposed YOLOv4 as a faster detection and classification approach compared to other traditional methods because of its single stage process for both detection and classification. However, exploring other detection and classification models could further demonstrate the effectiveness of this approach on the given dataset.

Kaushik et al. [79] introduced one of the earliest approaches of using YOLO model for mitotic nuclei detection and count. The result was compared to the state-of-the-art Faster R-CNN models [80] with various backbone algorithms. The ICPR-2012 dataset [81] was used for the experiment. The training set contained 35 images and the test set contained 15 images. Each of these images are of 2084 × 2084 pixels. Patches are generated from these images with size 512 × 512 pixels each with a stride 32. The evaluation metrics were precision, recall, F1 Score and time consumption (s). These metrics values for all these models are following – for FasterRCNN with ResNet101 Backbone: 0.856, 0.735, 0.790, 32.0; for FasterRCNN with InceptionNet Backbone: 0.764, 0.801, 0.782, 122.5; for FasterRCNN with NASNet Backbone: 0.781, 0.673, 0.723, 74.8; for FasterRCNN with MobileNet Backbone: 0.712, 0.514, 0.597, 4.1. The results clearly demonstrate that YOLO outperformed all versions of Faster R-CNN [80].

Yu et al. [82] applied YOLOv5 in uterine smooth muscle tumors (UMTs) as nuclei detectors. Three categories of UMTs are identified - leiomyoma (including specific subtypes), leiomyosarcoma, and smooth muscle tumors of uncertain malignant potential (STUMP). The dataset was collected from the Department of Pathology of Beijing Obstetrics and Gynecology Hospital from May 2016 to May 2021. Accurate counting of mitosis is important in differentiating between these three subtypes. YOLO architecture is used effectively in this regard with precision, recall, accuracy and F1 score are 0.938, 0.893, 0.913, and 0.915 respectively. ResNet models are used for further classification of UMT types. The experiment used 233 slides of leiomyosarcomas, 108 slides of leiomyomas, and 30 digital slides of STUMP with H&E staining.

Hemmatirad et al. [83] experimented on both YOLOv4 and U-Net for Glomeruli detection in kidney H & E-stained WSIs. They utilized two public datasets (http://aidpath.eu, https://www.kaggle.com/c/hubmap-kidneysegmentation/Overview) for training and a private dataset from the University of Michigan for validation. The comparative analysis revealed sensitivities and specificities, ranging from 45 % to 74 % and 98 %–94 %, for H & E-stained and PAS-stained images, respectively. Fine tuning on PAS-stained dataset demonstrated superior performance. However, this work does not include the usage of YOLO versions e.g., YOLOv5 along with reporting other metrics.

### 3.4. YOLO utilized as part of nuclei segmentation pipeline

In contrast to the previous research works, YOLO models were used as part of a broader pipeline for nuclei segmentation as well. Usually, the pipelines involve some segmentation models like U-Net or mask RCNN as segmentation models, as well as classification models, ranging from traditional machine learning models (SVM, KNN) to deep learning classifiers like CNNs and Transformers [9]. Table 4 summarizes the salient points of these approaches with detailed summaries. Bhausaheb et al. [84] adopted YOLO as a nuclei position locator. Classifier models, including SVM, KNN, CNN, Deep CNN, Canid-based deep CNN, Hoofed deer-based deep CNN, and Deer Canid-based deep CNN, were used for the final classification. The nuclei were categorized into classes such as non-tubule, tubule, non-tumor nuclei, tumor nuclei, apoptosis, and mitosis. Training and validation were conducted on BreCaHAD and breast histopathology images datasets [85]. The dataset contained 162 whole-mount slide images and a total of 277,524 number of patches out of those slides. Their results showed that Deer Canid optimization-based deep CNN attained the accuracy, precision, recall, and F1 measure values of 92.96 %, 94.34 %, 93.45 %, 92.89 %.

Nemati et al. [86] used YOLOv5 for cell mitosis detection. After the mitosis detection, fuzzy-based classifiers are used to better identify mitosis further. Three different fuzzy-based classifiers were used – fuzzy random forest (FRF) [87], fuzzy k-nearest neighbor (FKNN) [88], fuzzy min-max (FMM) [89]. The ICPR14 [66] dataset was used in this experiment with 1200 labeled and 496 unlabeled images. Here the experiments were performed in four stages. At first only YOLOv5 was evaluated. Then the other three combinations of models were tested one by one - YOLOv5 + FMM, YOLOv5 + FKNN, and YOLOv5 + FRF. YOLOv5 combined with FRF showed the best result outcome with precision, recall and F1-score values 0.895, 0.848 and 0.873 respectively. Where YOLOv5 with FKNN showed the results as 0.865, 0.752, and 0.805 respectively, YOLOv5 with FMM showed 0.822, 0.684, and 0.750 respectively and YOLOv5 without any fuzzy algorithm showed 0.818, 0.757, and 0.79. However other state-of-the-art algorithms could have been compared to demonstrate the effectiveness of the pipeline. Further other histopathology datasets can be tried to test if the assumption is provable to other data cohorts as well.

Rong et al. [90] tried to accelerate the nuclei segmentation and classification process and reduce the complexity. A novel algorithm, histology-based detection using YOLO (HD-YOLO), was developed to detect, segment and classify nucleus plus performed tumor microenvironment (TME) related feature extraction. Within HD-Yolo architecture, CSPDarkNet from YOLOv5 is used as backbone for image feature extraction. The bi-directional feature pyramid network (BiFPN) [91] was used to blend backbone feature maps at different resolutions. A new high-performance detector – YOLOX [43] was then used as detection head to localize and classify objects. Three different datasets are used in this experiment - Lung Adenocarcinoma, Liver Tissue and Breast Cancer dataset. The Lung Adenocarcinoma dataset has 127 patches (with 500 × 500 pixels each) extracted from 39 pathologic ROIs. A total of 105 patches extracted from 29 slides were allocated for training, while 12 patches from 5 slides were used for validation and 10 patches from the last 5 slides were used for testing purposes. The lung adenocarcinoma dataset (https://cdas.cancer.gov/nlst), liver tissue dataset (private) and breast cancer NuCLS dataset [69] were used. For the lung cancer datasets, in the evaluation of nuclei segmentation performance, HD-YOLO got superior results compared to other relevant algorithms such as HD-Staining, Mask R-CNN, and HoVer-Net [10]. The calculated metrics used were - accuracy, precision, recall, F1 score, mIoU, and processing time. Similarly, in the context of breast cancer, where metrics considered included accuracy, precision, recall, F1 score, and time, HD-YOLO outperformed a range of algorithms including Yolov7, Yolov6, Scaled-Yolov4, Deformable-DETR, EfficientDet, Mask R-CNN, and Cascade R-CNN.

Shi et al. [92] utilized the YOLO architecture for detection, incorporating additional CNN models like ResNet50 or InceptionNet as classifiers to identify and classify regions of interests (ROIs) in histopathology images, specifically targeting mitotic cells or nuclei. The classifiers achieved high accuracies of 98.8 % and 98.9 % for ResNet50 and InceptionNet, respectively. However, the authors did not provide any details about the dataset used and there is a lack of comparative analysis with other relevant detection and classification models.

Tyagi et al. [93] introduced a novel deep guided posterior regularization (DEGPR) framework, designed to enhance detector algorithms by guiding them to leverage discriminative features among cells. The DEGPR was applied with YOLOv5 and other relevant detector algorithms (Faster R-CNN [80] and EfficientDet) and compared with those models without DEGPR. The study utilized three distinct datasets—CoNSep [10], MoNuSAC [64], and a newly introduced dataset by the authors, MuCeD. The metrics used were precision, recall, mAP, MAE (mean absolute error) and MRE (mean relative error). From the given results, it is explicitly shown that the models with DEGPR performed better than the corresponding model without DEGPR. Overall, YOLOv5 has the best performances over the other two models with or without DEGPR.

Thelaya et al. [94] employed YOLOv5 as the nuclei detector in their pipeline, along with CNN models like SqueezeNet, ResNet, and EfficientNet as classifiers. The PanNuke dataset [62] was used for the experiment, however the paper lacks in providing metrics for the YOLO part evaluation. The highest successful nuclei detection rate is around 80 %. Patches were taken out from the bounding boxes, obtained using YOLOv5, around the nuclei. These patches were then fed into the classification models to receive better accuracy. YOLOv5 worked here as classification performance booster.

Çayır et al. [95] integrated YOLOv4 into their MITNET pipeline in a novel two-stage deep learning approach for mitosis recognition in whole slide images of breast cancer tissues. YOLOv4 was used as nuclei detector and a novel CNN architecture, based on VGG-11 [16] is used as the classifier part. The publicly available MIDOG and ATYPIA datasets were utilized in addition to their own private in-house dataset, comprising 139,124 annotated nuclei in 1749 patches with the size of 512 × 512 pixels extracted from 115 WSIs with the size of 87,780 × 109, 494 pixels. The YOLOv4 detection performance was promising with mAP of 0.88 at IoU = 0.5. However, the authors did not provide further experiment results with more details in metrics used and datasets applied. Also, other versions such as the YOLOv5 can be used to compare the results with YOLOv4.

Lin et al. [96] introduced a distinctive model, instance-YOLO, by combining YOLOv5 with U-Net. The approach involved ensembling two Hover-net [10] architectures to address class imbalance in colon nuclei dataset, followed by the application of the Instance-YOLO model for nuclei identification. The experimentation utilized the Lizard dataset [67].

Venugopal et al. [97] applied YOLOv5 as nuclei detector model on breast cancer datasets, namely the MITOS-ATYPIA-14 and MIDOG-2022. The outputs from YOLOv5 were bounding box coordinates which were fed into a classifier model - EfficientNetV2-S for binary classification – mitotic or non-mitotic cells. The YOLOv5 model was pre-trained using weights from the COCO dataset [29]. Evaluation metrics for YOLOv5 were separately calculated for each dataset. For MITOS-ATYPIA-14, the values were mAP@0.5: 0.933, Precision: 0.923, Recall: 0.928, and F1 score: 0.925. For MIDOG-2022, the metrics were 0.7812, 0.784, 0.7545, and 0.769, respectively. These results clearly indicate that YOLOv5 performs better in MITOS-ATYPIA-14. Exploring other nuclei detection models such as Mask R-CNN or Faster R-CNN could further showcase the effectiveness of the pipeline.

Zhu et al. [98] applied YOLOv3 as nuclei detector for cervical liquid-based thin-layer cell smear diagnosis where Xception and patch-based model were used for classification and U-Net was used for nuclei segmentation. The outputs from YOLOv3 were benchmarked against other detector algorithms, including faster R-CNN [80], SSD, and RETINANET. The evaluation metrics, mAP@0.5 and time consumption (ms), produced the following values for all the models are – YOLOv3: 82.1, 53; FASTER R-CNN: 85.6, 172; SSD: 80.9, 125 and RETINANET: 84.3, 198. However, the YOLOv3’s efficiency was found as 2.5–4 × higher than others.

YOLOv5 was applied as nuclei detector in the context of Barrett’s esophagus (BE) by Faghani et al. [99]. BE has a tendency to enhance with the expansion of dysplasia. Treatment for BE largely depends on type of dysplasia - nondysplastic BE (NDBE), low-grade dysplasia (LGD), and high-grade dysplasia (HGD). HGD is characterized by bigger nuclei sizes compared to LGD. Also, loss of cellular polarity, increased mitotic activity, and structural alterations including budding, branching, and cribriform formation are considered factors for the distinction between HGD, LGD and NDBE. YOLOv5 was used to find the coordinates of bounding boxes for the nuclei. The areas under the bounding boxes only then sent to a further classifier model, such as ResNet101. An H & E-stained private histopathological dataset is used with a total of 1494 number of slides. During this study a total of 542 patients were chosen – 164 with NDBE, 226 with LGD and 226 with HGD. The training set includes 368 slides, validation set includes 104 slides and test set includes 70 slides. For the whole ensemble model with YOLOV5 as a detector and ResNet as a classifier sensitivity and specificity for LGD were 81.3 % and 100 %, respectively, and greater than 90 % for NDBE and HGD. The F1 scores were 0.91 for NDBE, 0.90 for LGD, and 1.0 for HGD.

Zorgani et al. [100] ensembled YOLOv2 architecture with ResNet50 in their 2021 study for mitotic cell detection. The dataset used was breast cancer H & E slides from the ICPR 2012 competition [81]. The ResNet50 was used to extract deep features to leverage more insights from the images while YOLO provided fast and precise nuclei detection. The recall, precision and F1 score values for this model were 0.7765, 0.8049 and 0.7903 respectively. This result was compared with other studies as well proving this model superior to the others.

Ke et al. [101] utilized YOLO based architecture to detect bounding boxes over artifacts (including cell and nuclei) before further tissue structures restoration process. They used an artifact recognition (AR)-based approach to classify between normal tissue and restorable and un-restorable artifacts. Next, they used AR-CycleGAN to restore images obstructed by different artifacts. The artifact restoration is made smoother due to effective boundary boxes selection through YOLO.

Banerjee et al. [102] used YOLO as nuclei detector and to generate boundary boxes. SVM classifier was used to classify those boundary boxes into mitotic or not. Before nuclei detection, U-Net was used to segment nuclei as white compared to the background. The H & E data images were used from ICPR 2012 [81] and ICPR 2014 [66] challenges. The YOLO architecture used SqueezeNet as backbone instead of DarkNet. According to the paper, the use of SqueezeNet reduced the weights of model significantly.

## 4. Discussion and conclusion

Nuclei segmentation plays a vital role in the identification and classification of various cancer types. Pathologists depend on the microscopic examinations of tissue samples to identify abnormal patterns in cells which indicates various types of cancers. As the core components of cells, nuclei contain vital information about cellular morphology and characteristics. Thus, accurate nuclei segmentation is necessary to extract meaningful features that helps in distinguishing normal and malignant tissues. Using object detection techniques to delineate nuclei boundaries, pathologists can analyze nuclear morphology, size and distribution as crucial insights into underlying pathology. This process remarkably improves diagnostic accuracy besides making it faster than the traditional techniques. This further enriches researches on cancer types guiding customized treatment approaches and enhancing patient outcomes.

Since its first paper by Redmon et al. [11], YOLO model is growing in popularity as an important object detection tool with vast application areas. Over the last few years, YOLO has undergone many iterative improvements in order to transform the object detection process more accurate and faster. In the field of histopathology, where accurate identification and segmentation of nuclei play a crucial role in both diagnostics and research purposes, the integration of YOLO has shown potential in optimizing these procedures. The evolution of YOLO has not only transformed the nuclei segmentation process but has unlocked numerous possibilities in unveiling fresh milestones, challenges and innovations that significantly influence in increasing accuracy in nuclei segmentation.

Summarizing this review, we find that a total of 7 articles used YOLO directly as detector in their experiments (see Section 3.3). Here one or more YOLO versions were trained and evaluated with relevant nuclei H & E datasets. The experimental results were then compared with other deep learning models. The trend we observed in these works was to examine the state-of-the-art detector model, YOLO in the histopathology field to test its effectiveness in compared with traditional pathologists’ way. A total of 14 articles utilized YOLO architecture as part of their model pipeline (see Section 3.4.). In these works, typically YOLO was used as detector or segmentation model to find the boundary boxes out of the nuclei and one classifier is used to further classify the nuclei to predict the disease. We note that Bhausaheb et al. [84], Nemati et al. [86], Thelaya et al. [94], Venugopal et al. [97], Zhu et al. [98], and Faghani et al. [99] utilized YOLO along with separate classifier to detect, segment and classify nuclei. Several works showed that effectively changing YOLO architectures can increase the performance of overall pipeline. Zorgani et al. [100] used ResNet50 as backbone instead of DarkNet19 architecture to leverage the feature extraction efficiency of ResNet architecture which improves the overall nuclei detection performance significantly. Tyagi et al. [93] applied an additional auxiliary loss function, Posterior regularization, with the deep learning models like YOLOv5 to improve the feature learning process. Lin et al. [96] utilized the U-Net model’s object segmentation capability by combining U-Net architecture and YOLOv5. The combined model provides an improved version of nuclei detection and segmentation together. Rong et al. [90] used combination of YOLOX and YOLOv5 architectures to form new model, HD-YOLO. Ke et al. [101] used YOLO to find the boundary boxes over the nuclei before performing the images of artifact restoration process. YOLO was only evaluated as reference to compare the results with their own proposed models as seen in Ali et al. [60] and Huang et al. [61] who used YOLO as reference to benchmark their own proposed models.

We note that in some research works, YOLO was used as not a part of the pipeline but to compare with a proposed new model. Ali et al. [60] introduced CB-HVTNet, a model combining CNN and ViT with a channel boosting approach. Evaluated on LYSTO [103] and Nuclick [104] datasets for lymphocyte segmentation, CB-HVTNet outperformed YOLO. The F1-score and recalls for CB-HVTNet were 0.88 and 0.93, surpassing YOLO with values of 0.8 and 0.69. However, the paper did not reveal the used YOLO versions, which complicates the evaluation of advanced YOLO models’ efficiency on these datasets. Additionally, exploring other metrics like accuracy, precision, Dice and IoU scores could provide a more comprehensive comparison. Huang et al. [61] applied Mask R-CNN as nuclei detection model utilizing a private breast cancer dataset obtained from Case Western Reserve University. The evaluation was measured in terms of precision, recall and F1 score metrics, comparing Mask R-CNN with YOLO, single shot multibox detector (SSD) [24] [14], and Faster R-CNN [80]. The experiment showed that Mask R-CNN outperformed the other three models with precision, recall and F1 score values of 91.28, 87.68 and 89.44 respectively. In contrast, for YOLO the scores were only 37.64, 31.57 and 37.60 respectively. However, the earliest version of YOLO was used only. It is worth noting that exploring advanced versions of YOLO could potentially provide a more comprehensive demonstration of its capabilities in comparison.

Based on the review, we uncovered several challenges that remain within YOLO-based nuclei segmentation, which, if addressed, could lead to more robust and well-organized evaluations of state-of-the-art object detection models. For instance, certain works failed to disclose the precise results of running their respective YOLO models. Thelaya et al. [94], Ke et al. [101], Banerjee et al. [102], Çayır et al. [95] provided no evidence of YOLO model evaluation in their publications. Crucially some works omitted mention of the specific YOLO version and experimental hyperparameters used, thereby hindering understanding of the model’s inner architecture and feature learning map. This aspect also shows that there is a need for a common benchmark to evaluate the strength/weaknesses of different YOLO versions. Ali et al. [60], Bhausaheb et al. [84], Shi et al. [92], Ke et al. [101], Kaushik et al. [79], and Huang et al. [61] were among those that did not specify the YOLO version utilized. Some works relied on outdated versions of YOLO, overlooking the existence of more advanced state-of-the-art versions. Hemmatirad et al. [83] and Sreeraj et al. [73] did not use the updated YOLO versions in their experiments. Additionally, certain studies neglected to provide details about their datasets, making it challenging to assess them alongside other models. Tung et al. [72], Yu et al. [82], Shi et al. [92], Zhu et al. [98], and Faghani et al. [99], for instance, did not disclose links to their private datasets. Benchmarking the YOLO model or proposed models against other object detection models could have strengthened evaluations. Studies by Venugopal et al. [97], Shi et al. [92], Rong et al. [90], Nemati et al. [86], and Hemmatirad et al. [83] might have benefited from comparisons with additional models in this regard. Further, some papers lacked comprehensive metrics during YOLO model evaluation. Hemmatirad et al. [83] only assessed sensitivity and specificity, while metrics like Dice and IoU scores could have provided additional insights. Similarly, Shi et al. [92] solely relied on accuracy, overlooking the potential benefits of incorporating other metrics for a more comprehensive evaluation. Though YOLO has demonstrated significant potential in nuclei segmentation and detection in WSIs, several technical challenges exist, particularly with the earlier versions of YOLO, such as YOLOv3 and YOLOv4. One of the key limitations of early YOLO versions are their inability to effective handling of nuclei with varying sizes. This often leads to inaccuracies in detecting smaller or irregularly shaped nuclei. Newer versions of YOLO, such as YOLOv9 [105] and YOLOv10 [106], are fully equipped with dynamic task prioritization and, therefore, can balance object detection and segmentation tasks more effectively. YOLOv9 introduces a revised decoupled head enabling separation of classification and localization tasks resulting in more accurate bounding box predictions for nuclei of varying sizes and shapes. YOLOv10 introduced a more refined anchor-free approach to eliminate the need for predefined bounding box sizes. YOLOv10 also boasts an improved speed-accuracy tradeoff which makes it suitable for real-time applications, especially in nuclei segmentation for histopathology. YOLOv10 can capture better fine-grained details of spatial tissue data by introducing a novel feature extraction module without compromising the inference speed. In future, adapting these newer versions of YOLO, and integrating them with attention-based mechanisms, like transformers, could further enhance the performance in nuclei detection, particularly in cases where nuclei boundaries are ambiguous and overlapped.

During our review, we observed the relevance of applying YOLO in various research areas beyond nuclei detection in histopathology images. For instance, Prabhu et al. [107] utilized YOLO for the detection of keratin pearl, a critical feature in the detection of squamous cell carcinoma, from H & E-stained biopsy slides. Swiderska-Chadaj et al. [108] explored lymphocyte detection instead of nuclei detection using the YOLO model. Seesawad et al. [109] use YOLOv8 backbone for centroblast cell detection in H & E-stained WSIs which are used for grading malignant follicular lymphoma. Note that these and other studies have also demonstrated the efficient application of YOLO in images based on other staining protocols than the standard H & E-stained images. Swiderska-Chadaj et al. [108] tested their approach on immunohistochemical (IHC) images, Su et al. [110] and Narotamo et al. [111] utilized YOLO for nuclei detection in fluorescence-stained images. YOLO has been effectively used in tissue region detection in other medical imaging modalities as well. Tamang et al. [112] reviewed the use of YOLO models for colorectal cancer tissue detection across endoscopy and histopathology images.

In conclusion, our systematic review highlights the versatility and effectiveness of YOLO in automated nuclei segmentation within histopathology images. This further illustrates the broad applicability of YOLO across different histopathological datasets. As research continues to evolve, YOLO stands as a promising tool for improving accuracy, efficiency, and automation in nuclei segmentation and beyond.

## Figures and Tables

**Table 1:** Key features of YOLO versions.

YOLO Version	Strengths	Weaknesses	Characteristics
v1	Fast single-shot detection; grid-based approach	Struggles with detecting small objects; localization inaccuracies; lacks anchor boxes	24 convolutional layers, 2 fully connected layers, simple sum-squared error loss
v2	Improved detection with anchor boxes; better handling of object scales; supports 9000+ object categories	Still limited accuracy on small objects; performance affected by low-resolution images	Introduced DarkNet-19 backbone; uses multi-scale training and batch normalization
v3	Enhanced feature representation with deeper DarkNet-53 backbone; multi-scale predictions	Relatively larger model size compared to YOLOv2; slower than newer versions	Introduced Feature Pyramid Networks (FPN) for multi-scale detection
v4	High accuracy with CSPDarkNet53 backbone; new activation function (Mish); data augmentation (Mosaic, SAT); Complete IoU loss	Higher computational cost due to complex backbone; requires more resources	Incorporates PANet and SPP layers; achieves better AP on COCO dataset
v5	PyTorch implementation for better accessibility; auto-anchor optimization; smaller model sizes	Lack of peer-reviewed publication detailing its innovations	Introduced CIoU loss; supports MixUp and Mosaic data augmentation
Scaled YOLOv4	Allows scaling up or down to adapt to different hardware constraints; excellent AP with large-scale configurations	Reduced AP for smaller, faster configurations compared to specialized models	Introduced scaling techniques; supports TensorRT optimizations for high FPS
YOLOR	Unified architecture for feature extraction and prediction; high performance with explicit and implicit knowledge integration	Complex training process; relatively new and less explored in nuclei segmentation	High AP on COCO dataset with balanced speed; combines shallow and deep feature representations
YOLOX	Anchor-free design inspired by modern detectors (e.g., FCOS); decoupled heads for classification and regression	Requires careful training to optimize center sampling strategy	Combines YOLOv3’s efficiency with anchor-free innovation; high AP with relatively faster inference
v6	EfficientRep backbone for high parallelism; reduced head convolution layers for faster inference	Limited adoption in the research community	Achieved higher efficiency with reduced computational cost
v7	Introduced extended ELAN for improved gradient path learning; compound scaling for better resource allocation	Complex scaling may introduce overfitting for small datasets	Achieves a balance between speed and accuracy; state-of-the-art on COCO dataset
v8	Versatile tasks (segmentation, detection, pose estimation); anchor-free architecture; improved loss functions (CIoU, DFL)	Requires powerful GPUs for optimal performance due to higher complexity	Uses modified CSPLayer for high-level feature merging and contextual information integration

**Table 2:** Information of different nuclei segmentation datasets.

Dataset	Slides/Patches	Annotation Details	Tissue Types	Use Case
PanNuke	7904 images (256 × 256 pixels)	~200k labeled nuclei across 5 classes	19 Tissue types	Multiclass nuclei segmentation and classification across diverse cancer types
MIDOG	–	Annotations for mitotic and non-mitotic figures	Multiple cancer types	Mitosis detection for assessing tumor proliferation
MoNuSeg	32 images (1000 × 1000 pixels)	~22,000 nuclei with instance segmentation masks	Multiple organ tissues	Generalized nuclear segmentation for computational pathology
MoNuSac	Training: 20 images; Test: 14 images (1000 × 1000 pixels)	Segmentation and classification of 4 cell types (epithelial, lymphocyte, macrophage, neutrophil)	Breast, kidney, liver, prostate	Segmentation and classification of diverse nuclei types
Atypia	–	Nuclear atypia scoring (mitosis included)	Breast tissues	Scoring for low/moderate/high atypia in breast cancer
CoNSep	41 images (1000 × 1000 pixels)	Multiclass nuclei annotations	Colorectal adenocarcinoma	Segmentation of normal and tumor epithelial, inflammatory, and necrotic nuclei
Lizard	291 images (average size 1016 × 917 pixels)	~500,000 labeled nuclei across 6 cell classes	Colon tissue	Large-scale nuclei segmentation and classification in colonic tissue
NuCLS	~220,000 nuclei annotations	Expert-reviewed annotations	Breast tissues (TCGA dataset)	Large-scale segmentation and classification for breast cancer research

**Table 3:** Summary of YOLO-based nuclei segmentation methods wherein YOLO is used as the primary method.

Reference	Dataset	Imaging Modality	Summary	Results	YOLO Version
Tung et al [72, 72]	Private dataset from a regional teaching hospital in Taiwan. Link is not shared.	H&E	Nuclei segmentation from Gastric Cancer images using YOLOv4 and compare the results with pathologists’ manual results of detection.	Sensitivity 96.6 %, Specificity 89.6 %	YOLOv4
Sreeraj et al. (2021)[73]	MITOS-ATYPIA-14 Challenge dataset [66]	H&E	Breast cancer detection using Nuclei Atypia Scoring. In this process the cell nuclei structures are compared with cell structures. The nuclei detection is done by 4 YOLO models - YOLO-V3, tiny-YOLO, YOLO-V2 and YOLO-V1. These results are compared with each other’s.	Accuracies and precisions – YOLO-V3: 0.89, 0.87 Tiny-YOLO: 0.88, 0.85 YOLO-V2: 0.84, 0.8 YOLO-V1: 0.81, 0.77	YOLOv3, tiny-YOLO, YOLO-v2 and YOLOv1
Drioua et al [75,75]	BNS breast cancer dataset [76]	H&E	Detection of lesions from breast cancer slides using YOLO as cancer or not cancer.	YOLOv5 nuclei detection precision and recall are 0.86 and 0.77 respectively	YOLOv5
Nair et al [77, 77]	MITOS-ATYPIA grand challenge- 14 dataset [66]	H&E	Detection and classification of mitotic nuclei using YOLOv4. Classification categories – mitotic or non-mitotic. The images used were of two different staining process – Raw RGB images and Stain unmixed images [78].	F-score, recall and precision for raw RGB images were 0.73, 0.85 and 0.64 respectively and for stain unmixed images [78] were 0.65, 0.60 and 0.70 respectively.	YOLOv4
Kaushik et al [79,79]	ICPR-2012 dataset [81]	H&E	Mitotic counts using different Faster RCNN [80] models in breast cancer H&E images. Bench marking of those models shows that single Shot detectors such as YOLO and MobileNet perform poorly accuracy-wise but have the fastest running times.	Precision, Recall, F1Score, and Running time(s) for below mentioned models respectively are-FasterRCNN + ResNet101 Backbone: 0.856, 0.735, 0.790, 32.0 FasterRCNN + InceptionNet Backbone: 0.764, 0.801, 0.782, 122.5 FasterRCNN + NASNet Backbone: 0.781, 0.673, 0.723, 74.8 FasterRCNN + MobileNet Backbone: 0.712, 0.514, 0.597, 4.1 YOLO: 0.650, 0.772, 0.705, 5.8	NA
Yu et al [82, 82]	Private dataset collected from the Department of Pathology of Beijing Obstetrics and Gynecology Hospital from May 2016 to May 2021	H&E	Uterine smooth muscle tumors (UMTs) are diagnosed using nuclei detection using YOLOv5s. Three subtypes are detected based on counting nuclei. The three categories - leiomyoma (including specific subtypes), leiomyosarcoma, and smooth muscle tumors of uncertain malignant potential (STUMP)	Precision, Recall, Accuracy, and F1 Index - 0.938 0.893 0.913 0.915 respectively	YOLOv5
Hemmatirad et al [83,83]	Two public datasets – 1) http://aidpath.eu/, 2) https://www.kaggle.com/c/hubmap-kidneysegmentation /Overview One private dataset – collected from the University of Michigan.	H&E	Glomeruli and nuclei detection in kidney tissues using YOLOv4 and U-NET. Two different stained images are used H&E and PAS [113]. After fine tuning using PAS-stained images, the accuracies became much better.	Comparative analysis revealed sensitivities and specificities, ranging from 45 % to 74 % and 98 %–94 %, for H&E stained and PAS-stained images, respectively	YOLOv4

**Table 4:** Summary of research articles where YOLO is a part of the pipeline.

Reference	Dataset	Imaging Modality	Summary	Results	YOLO Version
Bhausaheb et al. (2023) [84]	BreCaHAD [85]	H&E	YOLO architecture was used for nuclei localization purpose in breast cancer dataset. Different classification models were used for final classification. The DL models were - SVM, KNN, CNN, Deep CNN, Canid-based deep CNN, Hoofed deer-based deep CNN, and Deer Canid-based deep CNN.	Accuracy, Precision, Recall, and F1 measure are for below classifiers – SVM: Dataset 1: 77.482, 82.433, 76.146, 77.603 Dataset 2: 78.500, 83.329, 76.960, 78.623 KNN [21]: Dataset 1: 85.156, 83.980, 88.014, 84.750 Dataset 2: 85.066, 84.329, 87.922, 84.660 NN: Dataset 1: 87.564, 88.940, 88.052, 87.494 Dataset 2: 88.754, 90.129, 89.241, 88.684 CNN: Dataset 1: 88.337, 89.712, 88.824, 88.266 Dataset 2: 89.159, 90.535, 90.805, 89.089 Deep CNN: Dataset 1: 88.723, 90.098, 90.898, 88.653 Dataset 2: 92.446, 93.822, 92.934, 92.376 Canid-deep CNN: Dataset 1: 92.581, 93.956, 93.068, 92.510 Dataset 2: 92.852, 94.227, 93.339, 92.781 Hoofed deer DCNN: Dataset 1: 92.195, 93.570, 92.682, 92.124 Dataset 2: 92.649, 94.024, 93.136, 92.579	NA
Nemati et al. (2023) [86]	MITOS-ATYPIA-14 Challenge dataset [66]	H&E	Classification of mitosis and non-mitosis using YOLO based segmentation and some machine learning algorithms (Fuzzy Random Forest [87], Fuzzy KNN [88] and Fuzzy Min Max [89] based classifiers.	Precisions, recalls and F1-scores respectively for each of the below combinations: YOLOv5: 0.818, 0.757, 0.79 YOLOv5 + Fuzzy Min-Max: 0.822, 0.684, 0.750 YOLOv5 + Fuzzy K-Nearest Neighbor: 0.865, 0.752, 0.805 YOLOv5 + Fuzzy Random Forest: 0.895, 0.848, 0.873	YOLOv5
Rong et al [90,90]	Lung adenicarcinoma dataset - https://cdas.cancer.gov/nlst Breast cancer dataset - (NUCLS) [69] Liver tissue dataset - private	H&E	A novel model architecture HD-YOLO, based on YOLOX and YOLO5 architectures, is invented for detection of nuclei and later segmentation. The model is later benchmarked with other similar YOLO architectures.	Accuracy Precision Recall F1 score Time per image for nucleus detection performance on the breast cancer – HD-Yolo: 0.8950 0.8042 0.9069 0.7992 0.8493 0.0089 Yolov7: 0.8723 0.8013 0.9065 0.7945 0.8468 0.0090 Yolov6: 0.8798 0.8133 0.9101 0.7963 0.8494 0.0088 Scaled-Yolov4: 0.8603 0.7977 0.8764 0.7810 0.8249 0.0123 FCOS: 0.7876 0.7574 0.8848 0.7556 0.8148 0.0381 Deformable-DETR: 0.7889 0.7620 0.8877 0.7588 0.8180 0.1420 EfficientDet: 0.8726 0.7950 0.8930 0.7936 0.8403 0.0951 Mask R-CNN: 0.8631 0.7881 0.8749 0.7828 0.8252 0.1462 Cascade R-CNN: 0.8230 0.7915 0.9103 0.7902 0.8460 0.1720	YOLOX, YOLOv5, YOLOv6, YOLOv7, Scaled-YOLOv4
Shi et al. [92]	Private dataset	H&E, CT, Mammograms	YOLO architecture is used to detect lesions in chest cancer from different types of datasets like H&E, mammograms and CT scan. To improve accuracies, other CNN architectures like - ResNet and InceptionNet are used as feature extractors.	Using YOLO as detector and ResNet and InceptionNet as backbone feature extractors the accuracies are 98.8 % and 98.9 % respectively.	NA
Zorgani et al. (2021) [100]	ICPR 2012 [81]	H&E	Ensembling YOLOv2 and ResNet50 architectures for mitotic cell detection for breast cancer images, provides better result than contemporary other studies.	Recall 0.7765 Precision 0.8049 F1 Score 0.7903	YOLOv2
Tyagi et al [93,93]	MuCeD (novel dataset, private), CoNSeP [10], and MoNuSAC [64]	H&E	Cell and nuclei segmentation and count using YOLOV5, Faster-RCNN and EfficientNet. A novel deep guided posterior regularization (DEGPR) framework is used for which the posterior distribution of a predictor mimics the data distribution for the given features. DeGPR implies PR (Posterior regularization) over two different features - implicit (learned features during training) and explicit (introduced by pathologists) features. DeGPR is applied with both the YOLO, Faster-RCNN and EfficientNet and the final results are benchmarked with the corresponding without DeGPR versions.	1) Precision, Recall, mAP, MAE IEL, MRE IEL, MAE Epith, MRE Epith for MuCeD dataset for each of the below models are - Yolov5 0.711 0.723 0.751 8.97 42.62 14.61 13.43 Yolov5 (DEGPR) 0.744 0.735 0.787 5.83 24.19 13.15 12.46 Faster-RCNN 0.592 0.436 0.496 11.85 50.05 27.50 24.93 Faster-RCNN (DEGPR) 0.646 0.468 0.541 9.61 31.64 26.50 23.60 EfficientDet 0.266 0.640 0.414 20.35,133.91 20.30 20.78 EfficientDet (DEGPR) 0.274 0.641 0.425 17.32 90.04 18.51 18.12 2) Precision Recall mAP MAE Inflm MAE Epith MAE Spindle MAE Avg for CoNSep dataset for each of the below models are - Yolov5 0.638 0.574 0.606 28.21 55.50 57.93 47.21 Yolov5 (DEGPR) 0.667 0.584 0.625 26.35 55.00 53.85 45.07 Faster-RCNN 0.490 0.208 0.342 64.71,227.93,198.29,163.64 Faster-RCNN (DEGPR) 0.571 0.331 0.451 51.93,151.28,163.00 122.07 EfficientDet 0.633 0.178 0.205 86.00 79.86,134.36,100.27 EfficientDet (DEGPR) 0.672 0.194 0.229 79.64 77.78,125.85 94.42 3) Precision Recall mAP MAEEpithelial MAELymphocyte MAENeutrophil MAEMacrophage for MoNuSAC dataset for each of the below models are - Yolov5 0.611 0.497 0.481 25.15 14.12 1.96 3.95 Yolov5 (DEGPR) 0.736 0.474 0.489 12.01 10.69 0.81 2.33 Faster-RCNN 0.570 0.310 0.405 19.52 23.48 1.0 3.38 Faster-RCNN (DeGPR) 0.643 0.370 0.473 19.81 22.44 0.82 3.02 EfficientDet 0.256 0.509 0.402 17.67 17.25 1.24 6.51 EfficientDet (DEGPR) 0.258 0.499 0.409 14.84 16.98 0.56 3.97	YOLOv5
Thelaya et al. (2022) [94]	PanNuke [62] for training and private “GDC dataset” for validation	H&E	YOLO is used for nuclei segmentation or localization. The classification tasks are done by models like EfficientNet, SqueezeNet and ResNet	From the paper it can be derived that the success rate decreases as the IoU threshold increases. The confusion matrix shows the classification results for nuclei categories - Neoplastic, Inflammatory, Connective/Soft, Dead, Epithelial.	YOLOv5
Çayır et al [95,95]	MIDOG [63], MITOS-ATYPIA-14 [66], private in-house dataset	H&E	New MITNET-det model and MITNET-rec model was proposed for mitosis detection and classification, where Scaled-YOLOv4 was used as nuclei detector. The result was compared with other DL models like – ResNet, DenseNet and EfficientNet.	MITNET-rec outperformed the other models with the metrics result values as precision 58.6 %, recall 82.9 %, and F1-score 68.7 %	Scaled-YOLOv4
Lin et al [96, 96]	Subset of Lizard [67]-DigestPath, CRAG, GlaS, CoNSeP and PanNuke	H&E	Colon nuclei identification by combining Separable-HoverNet [10] (two HoverNet models) and Instance-YOLOv5. YOLOv5 is combined with U-Net to get Instance-YOLOv5 model.	mPQ+ and r2 values are mentioned respectively below for HoverNet [31] [10] and the proposed model- HoverNet -> 0.296, −0.428 Proposed model -> 0.389, 0.599	YOLOv5
Venugopal et al. (2022) [97]	MITOS-ATYPIA-14 [66], MIDOG [63], and COCO dataset [29]	H&E	YOLOv5 was used for nuclei detector and a classifier EfficientNetV2-S was used to classify the mitotic and non-mitotic nuclei. The model is pre-trained with COCO dataset.	mAP@0.5, Precision, Recall and F1 score for detection using YOLOv5 for each dataset are below - For MITOS-ATYPIA-14 −0.933, 0.923, 0.928, 0.925 And for MIDOG-2022 - 0.7812, 0.784, 0.7545, 0.769	YOLOv5
Zhu et al [98, 98]	Private dataset	H&E	For cervical liquid-based thin-layer cell smear diagnosis, integration of three models were done – YOLOv3 for nuclei detection, Xception and Patch-based models to boost target classification, and U-net for nucleus segmentation. The YOLO detection result is benchmarked with other models FASTERR-CNNWFPN, SSD513, and RETINANET.	For binary class detection model YOLOv3 outperforms other selected models The result is below - mAP@0.5 (in percentage) and time consumption (ms) for each model respectively- FASTERR-CNNWFPN: 85.6, 172 SSD513: 80.9, 125 RETINANET: 84.3, 198 YOLOV3–608: 82.1, 53 For multi-class detection, YOLOv3 has mAP@0.5 value as 82.33 %	YOLOv3
Faghani et al [99,99]	Private dataset	H&E	Classification of nondysplastic BE (NDBE), low-grade dysplasia (LGD), and high-grade dysplasia (HGD) using histology slides utilizing YOLOv5 as nuclei detector and ResNet101 as further classification.	The ensemble model was used, sensitivity and specificity for LGD was 81.3 % and 100 %, respectively, and >90 % for NDBE and HGD. The overall positive predictive value and sensitivity metric (calculated as F1 score) was 0.91 for NDBE, 0.90 for LGD, and 1.0 for HGD	YOLOv5
Ke et al [101, 101]	MoNuSeg2018 [65]	H&E	A YOLO-based system to detect artifact bounding boxes before employing an AR-classifier to differentiate between normal tissue and restorable/unrestorable artifacts. Then AR-CycleGAN used to restore images obscured by artifacts, capitalizing on the accurate boundary box selection enabled by YOLO for smoother restoration.	No result for YOLO was provided	YOLOv4
Banerjee et al [102,102]	ICPR 2012 [81] and ICPR 2014 [66]	H&E	YOLO is used to detect nuclei. Instead of DarkNet, they used SqueezeNet within YOLO architecture. After getting the boundary boxes out of YOLO, classification is done (mitotic or not) using SVM algorithm.	No result for YOLO was provided	NA

## Data Availability

No data was used for the research described in the article.

## Appendix

**Table 5:** Abbreviations used in the paper

AI	Artificial Intelligence
AP	Average precision
AR	Artifact recognition
BE	Barrett’s esophagus
BiFPN	Bi-directional feature pyramid network
CIOU	Complete intersection over union
CNN	Convolutional neural network
CSPLayer	cross stage partial layer
DEGPR	Deep guided posterior regularization
DFL	Distribution focal loss
DL	Deep learning
ELAN	Efficient layer aggregation network
FCOS	Fully convolutional one-stage
FKNN	Fuzzy k-nearest neighbor
FN	False negative
FRF	Fuzzy random forest
FP	False positive
FPN	Feature pyramid network
GIoU	Generalized intersection over union
GPU	Graphical processing unit
GT	Ground truth
H & E	Hematoxylin and eosin
HGD	High-grade dysplasia
IHC	Immunohistochemistry
IoU	Intersection over union
KNN	k-nearest neighbor
LGD	Low-grade dysplasia
MAE	Mean absolute error
mAP	Mean average precision
ML	Machine learning
mPQ	Mean panoptic quality
MRE	Mean relative error
NDBE	Nondysplastic Barrett’s esophagus
NMS	Non-maximum suppression
NPV	Negative predictive value
PPV	Positive predictive value
R-CNN	Region-based convolutional neural network
ROI	Region of interest
SIoU	SCYLLA intersection over union
SPP	Spatial pyramid pooling
SSD	Single shot multibox detector
STUMP	Smooth muscle tumors of uncertain malignant potential
SVM	Support vector machine
TN	True negative
TP	True positive
UMTs	Uterine smooth muscle tumors
VFL	VariFocal loss
WSI	Whole slide image
YOLO	You only look once
YOLOR	You only learn one representation
